# In vitro protein tyrosine phosphatase 1B inhibition and antioxidant property of different onion peel cultivars: A comparative study

**DOI:** 10.1002/fsn3.863

**Published:** 2018-12-15

**Authors:** Su Jin Yang, Pradeep Paudel, Srijan Shrestha, Su Hui Seong, Hyun Ah Jung, Jae Sue Choi

**Affiliations:** ^1^ Department of Food and Life Science Pukyong National University Busan Korea; ^2^ Department of Food Science and Human Nutrition Chonbuk National University Jeonju Korea

**Keywords:** antidiabetes, antioxidants, insulin‐resistant HepG2 cells, Onion peel, PTP1B

## Abstract

The aim of the present study was a comparative investigation of water and 70% ethanol extracts derived from yellow and red onion (*Allium cepa* L.) peels against diabetes and diabetic complications. The total phenolic contents (TPCs) and total flavonoid contents (TFCs) of each cultivar, measured to assess phytochemical characteristics, showed a direct correlation with the in vitro antioxidant effects. Among the two captives, the yellow onion peel extract showed higher antioxidant activity than red one. However, all extracts exhibited significant protein tyrosine phosphatase 1B (PTP1B) inhibitory activity (IC_50_; 0.30–0.86 μg/ml), showing water extracts more potent (IC_50_; approximately 0.3 μg/mL), than the 70% ethanol extracts (IC_50_; approximately 0.8 μg/ml). Similarly, in insulin‐resistant HepG2 cells, all extracts enhanced the glucose uptake and reduced the expression of PTP1B in a concentration‐dependent manner, water extract displaying better activity. Our results overall suggest that in vitro antioxidant and antidiabetic potentials vary among red and yellow cultivars and extracting solvents, which could therefore be a promising strategy to prevent diabetes and associated complications.

## 1 INTRODUCTION

Diabetes mellitus (DM) is the most common form of metabolic disorder that damages our various organs such as heart, kidneys, blood vessels, nerves, and eyes, leading to lifelong disability and premature death. Insulin resistance with an inadequate insulin secretory response is the etiology of type 2 diabetes mellitus (T2DM; Umar, Ahmed, Muhammad, Dogarai, & Soad, 2010). It is considered as one of the most attentive chronic diseases of the recent time due to its high prevalence and significant social and economic consequences. It is predicted that more than 415 million people are suffering from diabetes in 2015. Among them, approximately 90% of the people are diagnosed with T2DM (Sun et al., 2017). Extended research on diabetes has discovered many synthetic drugs against diabetes. Though developed therapies are able to reverse health issues/complications related to diabetes, they lead to various side effects. For many years in traditional folk medicine, diabetes and other fatal diseases have been treated orally with the variety of plant extracts. Till date, more than 1,200 plant species with antidiabetic properties have been reported (Habeck, 2003; Said et al., 2008). Nowadays, metformin is the most popular drug for DM, which was discovered with reference to biguanide compound isolated from *French lilac* (Oubre, Carlson, King, & Reaven, 1997). The selected plant could also be a potential candidate for this aim.

Onion (*Allium cepa* L.), which is consumed fresh as well as processed, is one of the most important vegetables worldwide. It belongs to the Alliaceae family and is biennial. It is commercially produced as an annual vegetable. It may differ greatly in color of outer scales (yellow, red, and white) and bulb shape (Slimestad, Fossen, & Vågen, 2007). Many studies suggest that regular consumption of onion helps to decrease the risk of several abnormalities such as neurodegenerative disorder, cancer, cataract formation, ulcer development, osteoporosis, and cardiovascular diseases (Singh et al., 2009). Onion contains various biologically active molecules such as phenolic acids, flavonoids, cepaenes, thiosulfinates, and anthocyanins (Goldman, Kopelberg, Debaene, & Schwartz, 1996). Further, flavonoids have shown other biological activity such as inhibition of plasma aggregation and cyclooxygenase (COX) activity; histamine release and slow‐reacting substance of anaphylaxis (SRS‐A) inhibition; and antibacterial, antiviral, anti‐inflammatory, and antiallergic effects (Hope, Welton, Fiedler‐Nagy, Batula‐Bernardo, & Coffey, 1983). There have been various studies regarding the onion having the high level of flavonols (Hertog, Feskens, Kromhout, Hollman, & Katan, 1993; Suh, Lee, Cho, Kim, & Chung, 1999). But unfortunately, onion peel is considered as waste and more than 500,000 tons of onion waste is produced annually in the European Union alone (Benítez et al., 2011). It includes skin, outer layers, roots, and stalks. Due to its aroma and rapid development of phytopathogenic agents, it cannot be used as fodder as well as organic fertilizer. So they are dumped. Therefore, a possible solution could be the use of waste as a source of food ingredients as onion skin contains a significant amount of flavonoids than the edible portion by about 2–10 g/kg (Suh et al., 1999). In a study conducted to evaluate the antidiabetic effect of onion peel extract (Jung, Lim, Moon, Kim, & Kwon, 2011), 60% ethanol extract of onion peel ameliorated hyperglycemia and insulin resistance in high‐fat diet/streptozotocin‐induced diabetic rats via alleviating metabolic dysregulation of free fatty acids, suppressing oxidative stress, and upregulating peripheral glucose uptake. Similarly, a study by Lee et al. (2008) suggested that onion skin is effective in controlling hyperglycemia via α‐glucosidase inhibition. In addition, ethanol extract of onion peel improved exaggerated postprandial spikes in blood glucose and glucose homeostasis by inhibiting intestinal sucrase and thus delaying carbohydrate absorption (Kim, Jo, Kwon, & Hwang, 2011). Though ample of studies concluded the antidiabetic potentials of onion peel extract in vitro and in vivo, there are limited papers on comparative study on different onion cultivars. The composition of onion varies with cultivar, stages of maturation, environment, agronomic conditions, storage time, and bulb part (Abayomi & Terry, 2009; Downes, Chope, & Terry, 2010). So it is essential to investigate the antidiabetic and antioxidant activity of onion peel (cultivars) to include as a possible food ingredient.

In the present study, we investigated the antidiabetic activity of 70% ethanol and water extracts from the peel of *Allium cepa* red (RE) and yellow (YW) cultivar via assays for the inhibition of protein tyrosine phosphatase 1B (PTP1B), α‐glucosidase, and advanced glycation end products (AGEs). In addition, antioxidant activity was evaluated via 1,1‐diphenyl‐2‐picrylhydrazyl (DPPH) and 2,2′‐azino‐bis‐(3‐ethylbenzothiazoline‐6‐sulfonic acid) (ABTS) radical scavenging activity, and insulin‐sensitizing property via 2‐NBDG glucose uptake in insulin‐resistant HepG2 cells.

## 2 MATERIALS AND METHODS

### 2.1 Chemicals and reagents

Protein tyrosine phosphatase 1B (PTP1B; human recombinant) was purchased from Biomol International LP (Plymouth Meeting, PA), dithiothreitol (DTT) was purchased from Bio‐Rad Laboratories (Hercules, CA), and sodium azide was purchased from Junsei Chemical Co. (Tokyo, Japan). Yeast α‐glucosidase, *p*‐nitrophenyl phosphate (*p*NPP), *p*‐nitrophenyl α‐D‐glucopyranoside (*p*NPG), ethylenediaminetetraacetic acid (EDTA), β‐nicotinamide adenine dinucleotide phosphate (NADPH), Folin–Ciocalteu reagent, gallic acid, Trolox, ascorbic acid, ursolic acid, acarbose, quercetin, DPPH, l‐penicillamine, dl‐glyceraldehyde, bovine serum albumin (BSA), sodium bicarbonate, dimethyl sulfoxide (DMSO), D‐(−)‐fructose, D‐(+)‐glucose, aminoguanidine, and rosiglitazone were purchased from Sigma‐Aldrich (St. Louis, MO). Fetal bovine serum (FBS), minimum essential medium (MEM), sodium pyruvate, penicillin–streptomycin, nonessential amino acids, and the fluorescent D‐glucose analog and glucose tracer 2‐[N‐(7‐nitrobenz‐2‐oxa‐1, 3‐diazol‐4‐yl) amino]‐2‐deoxy‐D‐glucose (2‐NBDG) were purchased from Life Technologies (Carlsbad, CA). Human insulin was purchased from Eli Lilly (Fegersheim, France). All other chemicals and solvents used were purchased from E. Merck, Fluka, and Sigma‐Aldrich.

### 2.2 Plant materials

Cultivating conditions: Cultivating conditions of onion depend on the desired part, that is either whole part as green vegetables or bulbs. Onion bulbs are cultivated using either seeds or small bulbs itself in a raised bed of loamy soil in the spring and harvested in the fall after their tops begin to die back or leaves turn to yellowish‐brown, indicating maturity. Temperature of 21–26°C favors rapid growth of bulbs. Once their tops begin to die, they are de‐soiled, and bulbs are separated, cleaned, and air‐dried.

The yellow and red onion cultivars were purchased in November 2015 from a local retailer and authenticated by Prof. Jae Sue Choi (Pukyong National University, Busan, Republic of Korea). Onions with similar sizes were sorted out and peeled off, and the peels were further air‐dried and quantified. Tentatively, around 200 red onions and around 150 yellow onions were peeled off. While sorting onions according to size, we acquired slight difference in onion number. A voucher specimen (#201511) was deposited in the authorized laboratory.

### 2.3 Preparation of samples

The peels of onion were dried under shade and coarsely powdered for extraction. Dried red onion peel (110 g) and dried yellow onion peel (70 g) were extracted with 5 L of 70% ethanol (EtOH) for 3 times to get 15.93 g of red onion peel 70% ethanol extract (RE) and 6.24 g of yellow onion peel 70% ethanol extract (YE), respectively. Similarly, dried red onion peel (110 g) and dried yellow onion peel (70 g) were extracted with 5 L of water for 3 times to get 23.25 g of red onion peel water extract (RW) and 7.41 g of yellow onion peel water extract (YW).

### 2.4 Determination of total phenolic content

The total phenolic content (TPC) of each of 70% ethanol and water extracts of dried peel of red and yellow onion was determined using the Folin–Ciocalteu reagent as described previously (Iqbal & Bhanger, 2006). The results were recorded as mg of gallic acid equivalent (GAE) per g of extract. The GAE values were expressed as mean ± *SEM* of triplicate experiments.

### 2.5 Determination of total flavonoid content

The total flavonoid content (TFC) of samples was measured by the aluminum chloride colorimetric method as described previously (Iqbal & Bhanger, 2006).

### 2.6 1,1‐Diphenyl‐2‐picrylhydrazyl radical scavenging assay

The 1,1‐Diphenyl‐2‐picrylhydrazyl (DPPH) radical scavenging activity of the samples was evaluated using method described previously using *L*‐ascorbic acid as the positive control (Iqbal & Bhanger, 2006).

### 2.7 2,2′‐Azino‐bis‐(3‐ethylbenzothiazoline‐6‐sulfonic acid) free radical scavenging assay

2,2′‐Azino‐bis‐(3‐ethylbenzothiazoline‐6‐sulfonic acid) (ABTS) radical scavenging assay of the samples was performed using method described previously (Ali, Jung, Jannat, Jung, & Choi, 2016).

### 2.8 Protein tyrosine phosphate 1B inhibitory assay

Protein tyrosine phosphate 1B (PTP1B) inhibitory assay of the samples was determined using method as mentioned previously (Paudel et al., 2018). The PTP1B inhibitory activity of each sample was expressed in terms of IC_50_ (μg/ml) and expressed as mean ± *SEM* of triplicate experiments.

### 2.9 α‐Glucosidase inhibitory assay

α‐Glucosidase inhibitory assay of the samples was determined using method as mentioned previously (Jung, Paudel, Seong, Min, & Choi, 2017). α‐Glucosidase inhibitory activity of each sample was expressed in terms of IC_50_ (μg/ml) and expressed as mean ± *SEM* of triplicate experiments.

### 2.10 Advanced glycation end product formation inhibitory assay

Advanced glycation end products (AGEs) formation inhibitory assay of different samples was determined as described earlier (Shrestha et al., 2018).

### 2.11 Cell culture, MTT assay, and insulin resistance induction

Human hepatocarcinoma (HepG2) cells were purchased from the American Type Culture Collection (HB‐8065; Manassas, VA). Cells were maintained at 37°C in a humidified atmosphere with 5% CO_2_ in 10% FBS MEM. Cytotoxicity of extracts was evaluated using the MTT assay (Mosmann, 1983). For developing insulin‐resistant HepG2 cell model, method by Liu et al. was followed (Liu et al., 2015). Rest of the experimental conditions and procedures were similar to those reported in our previous paper (Bhakta et al., 2017).

### 2.12 Glucose uptake assay

The fluorescent D‐glucose analog 2‐[N‐(7‐nitrobenz‐2‐oxa‐1, 3‐diazol‐4‐yl) amino]‐2‐deoxyglucose (2‐NBDG) was employed to evaluate glucose uptake rate in insulin‐resistant HepG2. Experimental conditions and steps followed to evaluate glucose uptake were same as previously described (Paudel et al., 2018). Rosiglitazone (10 μmol/L) was used as a reference drug.

### 2.13 Preparation of cell lysates and western blot analysis

Standard protocol was followed to prepare lysates of insulin‐resistant HepG2 cells using sample buffer and PMSF. Fifty micrograms of protein, once quantified by modified Bradford protein assay kit, was separated using a 12% sodium dodecyl sulfate–polyacrylamide gel electrophoresis (Bio‐Rad, Hercules, CA). The polyvinylidene difluoride (PVDF) membranes were incubated overnight on a shaker at 4°C with primary antibody prepared in 5% skim milk and visualized on X‐ray film after incubating PVDF membranes with secondary antibody for 2 hr at room temperature. Band intensities were quantitated using CS analyzer software (Atto Corp., Tokyo, Japan).

### 2.14 Statistical analysis

The results are presented as the mean ± standard error of the mean (*SEM*) following one‐way ANOVA and Duncan's test (Systat Inc., Evanston, IL). A *p*‐value <0.05 was considered significant for the differences.

## 3 RESULTS

### 3.1 Yield (%), total phenolic content, and total flavonoid content of onion extracts

Red and yellow onions were extracted with both 70% EtOH and water to obtain respective extracts. As shown in Table 1, the yield percentage (%) of RE, YE, RW, and YW were 14.48, 8.91, 21.14, and 10.58, respectively. Folin–Ciocalteu reagent and AlCl_3_ were used to determine the TPCs and TFCs, respectively, in the 70% ethanol and water extracts of onions (Table 1). The TPC and TFC results were recorded as mg of GAE per gram of dried extract and mg of QE per gram of dried extract, respectively. Both extracts exhibited high levels of TPC and TFC, being 70% EtOH extracts at the top in both cultivars. Between 70% EtOH extracts, yellow onion showed higher values of TPC (335.14) and TFC (214.42) than red onion (TPC [233.40] and TFC [181.86]). The values of TPC and TFC in water extracts were lower than the 70% EtOH extracts. Between water extracts, the values of TPC were slightly higher in yellow onion than red onion, which were 142.47 and 112.09, respectively. Whereas, the values of TFC among water extracts vary significantly among cultivars, which were 142.09 and 31.96 for yellow and red onions, respectively. Overall, the values of TPC and TFC followed a pattern of YE > RE > YW > RW.

**Table 1:** Total phenolic and flavonoid contents of onion extracts (mean ± *SEM*,* n* = 3)

Extracts	Yield (%)^a^	Total phenolic content (mg GAE/g of sample)	Total flavonoid content (mg QE/g of sample)
70% EtOH extracts			
Red onion	14.48	233.40 ± 0.58^c^	181.86 ± 0.01^c^
Yellow onion	8.91	335.14 ± 0.29^b^^,^ c	214.42 ± 0.21^b^^,^ c
Water extracts			
Red onion	21.14	112.09 ± 0.01	31.96 ± 0.21
Yellow onion	10.58	142.47 ± 0.29^b^	142.09 ± 0.21^b^

a Yield (%): The yield (w/w) percentage of the 70% EtOH and water extracts from two onion cultivars.

b Significant difference between red and yellow onions (*p *<* *0.05).

c Significant difference between 70% EtOH and water extracts of onions (*p *<* *0.05).

### 3.2 1,1‐Diphenyl‐2‐picrylhydrazyl radical scavenging activity of onion extracts

**Figure 1:**
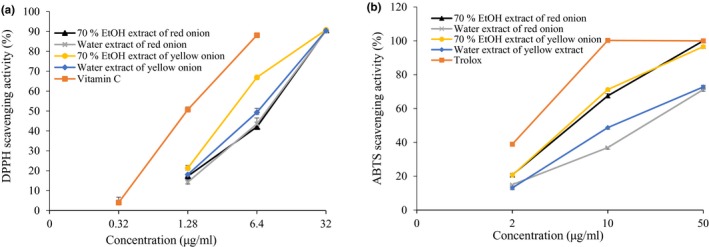
The ability of 70% EtOH and water extracts from onion to scavenge 1,1‐diphenyl‐2‐picrylhydrazyl (DPPH) (a) and 2,2′‐azino‐bis‐(3‐ethylbenzothiazoline‐6‐sulfonic acid) (ABTS) radical (b). All values were expressed as the mean ± *SEM* of triplicate experiments

To evaluate the antioxidant ability of onion peel of red and yellow cultivars, the 70% EtOH and water extracts were tested for in vitro 1,1‐Diphenyl‐2‐picrylhydrazyl (DPPH) radical scavenging activity. Based on the formation of the DPPH‐H nonradical form in the presence of hydrogen‐donating antioxidants in the extracts, the DPPH radical scavenging activity was determined. The DPPH radical scavenging activity of 70% EtOH and water extracts of red and yellow onion was tested at different concentrations using L‐ascorbic acid as positive standard. The results are demonstrated in Table 2 and Figure 1a. The extracts at a concentration of 6.4 μg/ml showed a range of 42%–66% DPPH radical scavenging activity in the concentration‐dependent manner, and the IC_50_ value ranged from 4.50 ± 0.06 to 10.60 ± 0.18 μg/ml. YE showed an IC_50_ value of 4.50 ± 0.06 μg/ml, which was the best among other extracts with 66.97% DPPH radical scavenging activity at 6.4 μg/ml. Ascorbic acid was used as positive control, which had the IC_50_ value of 1.27 ± 0.01 μg/ml. In addition, water extract of yellow onion demonstrated potential DPPH scavenging activity with an IC_50_ value of 6.77 ± 1.27 μg/ml, which was also better than the both extracts of red onion. Further, the IC_50_ values of RE and RW were found to be 10.60 ± 0.18 μg/ml and 9.86 ± 1.40 μg/ml.

**Table 2:** Antioxidant and antidiabetic activity of the 70% ethanol and water extracts from onion (mean ± SEM, *n* = 3)

Samples	IC_50_ (μg/ml, Mean ± *SEM*)^a^				
	DPPH	ABTS	PTP1B	α‐Glucosidase	AGEs
70% EtOH extracts					
Red onion	10.60 ± 0.18^e^	7.00 ± 0.20^d^	0.76 ± 0.17^c^	5.76 ± 0.03^bc^	12.79 ± 0.22^b^
Yellow onion	4.50 ± 0.06^c^	6.64 ± 0.03^c^	0.86 ± 0.04^c^	3.90 ± 0.08^b^	12.25 ± 1.35^b^
Water extracts					
Red onion	9.86 ± 1.40^e^	29.04 ± 0.11^f^	0.33 ± 0.01^b^	8.99 ± 0.56^c^	51.91 ± 1.53^d^
Yellow onion	6.77 ± 1.27^d^	12.03 ± 0.23^e^	0.30 ± 0.08^b^	5.44 ± 0.06^bc^	25.17 ± 1.75^c^
Positive controls					
Ascorbic acid	1.27 ± 0.01^b^				
Trolox		2.76 ± 0.05^b^			
Ursolic acid			3.40 ± 0.34^d^		
Acarbose				70.17 ± 4.96^d^	
Aminoguanidine					58.47 ± 0.17^e^

a The 50% inhibitory concentration (IC_50_) values (μg/ml) were calculated from a log dose concentration–inhibition curve and expressed as mean ± *SEM* of triplicate experiments.

Mean with different superscripts letters (b–f) are significantly different with Duncan's test at *p *<* *0.05.

### 3.3 2,2′‐Azino‐bis‐(3‐ethylbenzothiazoline‐6‐sulfonic acid) (ABTS) free radical scavenging activity of onion extracts

Table 2 and Figure 1b shows the 2,2′‐Azino‐bis‐(3‐ethylbenzothiazoline‐6‐sulfonic acid) (ABTS) radical scavenging activity of onion. Overall, all extracts showed good ABTS radical scavenging activity. Comparatively, 70% EtOH extracts showed better activity than the water extracts in both cultivars. At 10 μg/ml, YE showed 71.24% ABTS radical scavenging activity with an IC_50_ value of 6.64 ± 0.03 μg/ml, whereas RE showed 67.51% with an IC_50_ value of 7.00 ± 0.02 μg/ml, compared to the positive standard Trolox (2.76 ± 0.05 μg/ml). In addition, water extracts showed IC_50_ value of 12.03 ± 0.23 and 29.04 ± 0.11 μg/ml for yellow and red onion cultivars, respectively.

### 3.4 Inhibitory activities of onion extracts on protein tyrosine phosphatase 1B

**Figure 2:**
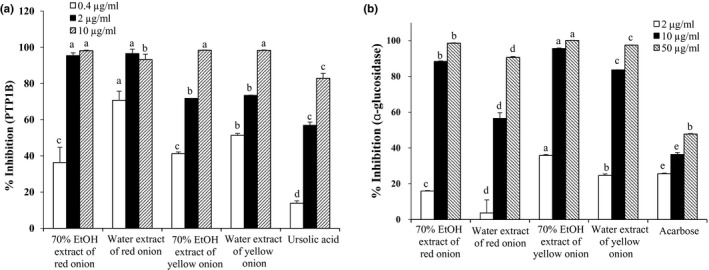
Inhibition profile of protein tyrosine phosphatase 1B (PTP1B) (a) and α‐glucosidase (b) by 70% EtOH and water extracts from onion. All values were expressed as the mean ± *SEM* of triplicate experiments. Means with different letters are significantly different with Duncan's test at *p *<* *0.05

The bioactivity of extracts on PTP1B inhibition was examined in vitro using *p*NPP substrate. The results of our study showed effective inhibition of PTP1B by the EtOH and water extracts of both red and yellow cultivars of onion, at concentrations of 0.4–10 μg/ml. The water extracts showed the highest activity, and at concentrations of 0.4, 2, and 10 μg/ml, the inhibition rates were in a range of 51.37%–98.34% (Figure 2a). In addition, YW with an IC_50_ of 0.30 ± 0.08 μg/ml was 10 times more potent than the positive control ursolic acid (3.40 ± 0.34 μg/ml) and 3 times more potent than the YE extract (0.86 ± 0.04 μg/ml). Further, RW also showed good inhibitory activity with an IC_50_ of 0.33 ± 0.01 μg/ml, which was similar to the YW, but had higher activity than the YW at lower concentrations (0.4 and 2 μg/ml).

### 3.5 Inhibitory activities of onion extracts on α‐glucosidase

All extracts significantly inhibited the α‐glucosidase in a concentration‐dependent manner (Figure 2b). The activity of RE, RW, YE, and YW was considerably higher with IC_50_ value of 5.76 ± 0.03, 8.99 ± 0.56, 3.90 ± 0.08, and 5.44 ± 0.06 μg/ml, respectively, than the positive control acarbose (IC_50_; 70.17 ± 4.96 μg/ml; Table 2). At 10 μg/ml, YE extract produced highest inhibition of 95.55%, while RE and YW extracts showed similar activity with 88.38% and 83.64% of inhibition.

### 3.6 Inhibitory activities of onion extracts on advanced glycation end products

**Figure 3:**
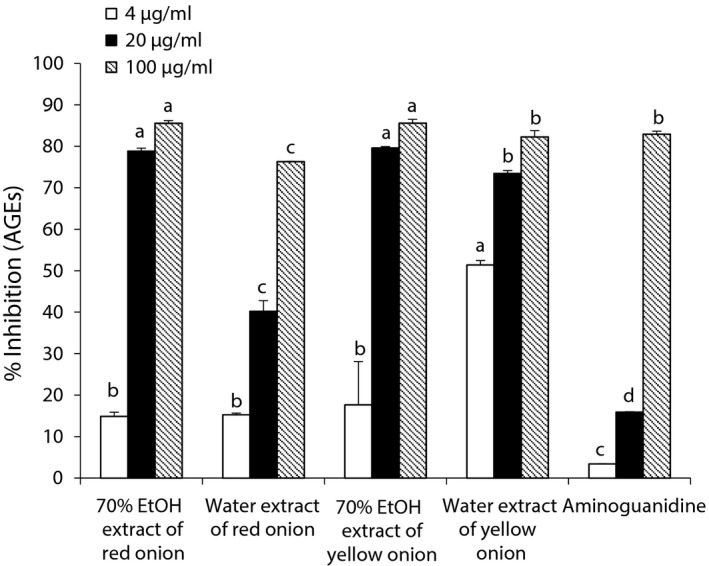
Inhibition profile of advanced glycation end products (AGEs) by 70% EtOH and water extracts from onion. All values were expressed as the mean ± *SEM* of triplicate experiments. Means with different letters are significantly different with Duncan's test at *p *<* *0.05

Table 2 and Figure 3 demonstrated the advanced glycation end products (AGE) inhibitory activity of different extracts. From the data, it can be clearly seen that 70% EtOH extract had better inhibitory activity than the water extract. At 20 μg/ml, 70% ethanol extracts showed approx. 79% inhibition in a concentration‐dependent manner with the IC_50_ value of 12.25 ± 1.35 and 12.79 ± 0.22 μg/ml for yellow and red onions, respectively. Also, the YW and RW indicated good inhibition with IC_50_ values of 25.17 ± 1.75 and 51.91 ± 1.53 μg/ml, respectively, compared to aminoguanidine (IC_50_; 58.47 ± 0.17 μg/ml).

### 3.7 Evaluation of cytotoxicity in HepG2 cells

**Figure 4:**
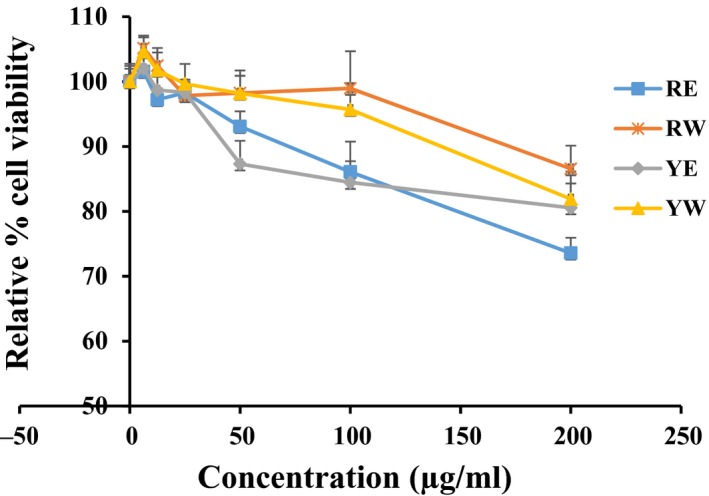
Effects of 70% EtOH and water extracts from onion on cell viability in HepG2 cells measured by MTT assay. HepG2 cells were treated with the indicated concentrations (0, 6.25, 12.5, 25, 50, 100, and 200 μg/ml) of extracts for 24 hr followed by 2‐hr MTT treatment (50 μg/ml). Data shown represent means ± *SD* of triplicate experiments

In order to find out the nontoxic concentration in HepG2 cells, we evaluated the cell viability using an MTT assay. As shown in Figure 4, water extract of both onion cultivars (RW and YW) showed no toxicity up to 100 μg/ml concentration. However, the 70% EtOH extract at 100 μg/ml showed 20% reduction in cell viability compared to normal control group.

### 3.8 Glucose uptake potentials in insulin‐resistant HepG2 cells

**Figure 5:**
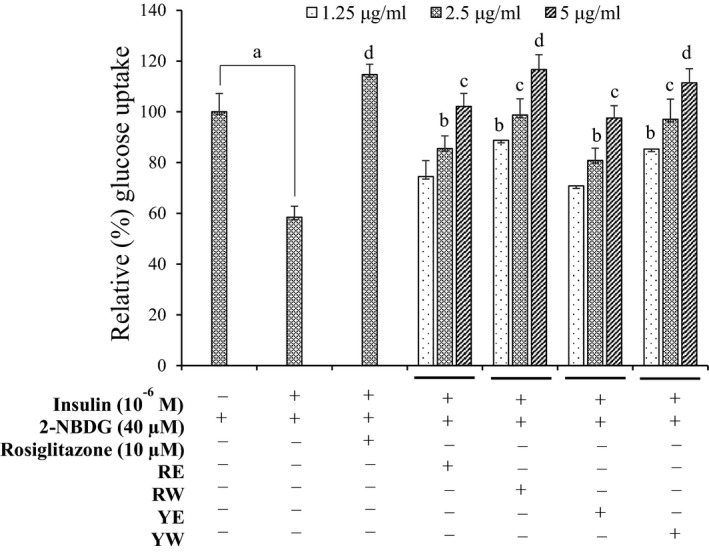
Effects of 70% EtOH and water extracts from onion on insulin‐stimulated glucose uptake in insulin‐resistant HepG2 cells as measured by 2‐[N‐(7‐nitrobenz‐2‐oxa‐1, 3‐diazol‐4‐yl) amino]‐2‐deoxyglucose (2‐NBDG) method. Data shown represent means ± *SD* of triplicate experiments. ^a^ *p* < 0.001 indicates significant differences from the control group; ^b^ *p* < 0.05, ^c^ *p* < 0.01 and ^d^ *p* < 0.001 indicate significant differences from the insulin‐resistant group

Considering the potent activity of extracts against PTP1B and wondering whether these extracts have ability to enhance glucose uptake, a 2‐NBDG uptake assay was performed in insulin‐resistant HepG2 cells. As shown in Figure 5, both solvent extracts of each type of cultivar showed a concentration‐dependent glucose uptake activity. However, when compared to the water with ethanol extracts of each onion cultivar, water extract of both cultivars showed potent activity. The percentage of glucose uptake at 1.25 μg/ml was around 70% for 70% EtOH extract of both cultivars; however, at the same concentration, water extract showed about 90% uptake.

### 3.9 PTP1B expression levels in insulin‐resistant HepG2 cells

**Figure 6:**
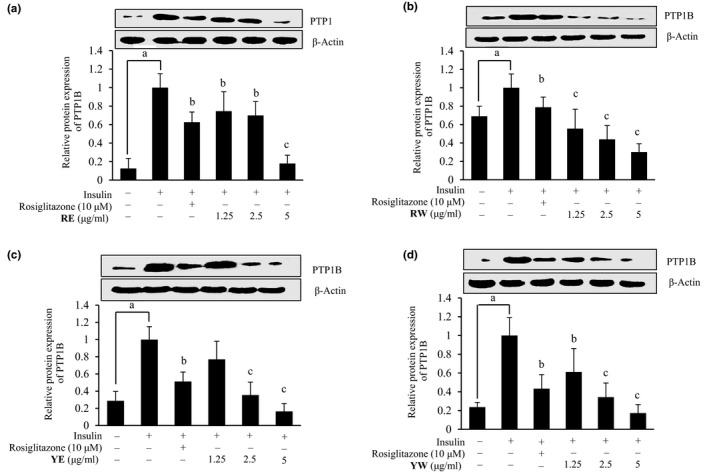
Effects of 70% EtOH and water extracts from onion on protein tyrosine phosphatase 1B (PTP1B) expression level in insulin‐resistant HepG2 cells. Western blotting was performed, and protein band intensities were quantified by densitometric analysis. Upper panels display representative blots. Equal protein loading was ensured and normalized against β‐actin levels. Values are the mean ± *SD* of three independent experiments; ^a^ *p* < 0.001 indicates significant differences from the control group; ^b^ *p* < 0.01 and ^c^ *p *< 0.001 indicate significant differences from the 10^−6^ M insulin‐treated control group

Protein tyrosine phosphatase 1B (PTP1B) regulates insulin signaling negatively, and its increased activity and expression are implicated in the pathogenesis of insulin resistance. In order to confirm whether the onion extracts modulate the PTP1B expression, insulin‐resistant HepG2 cells were treated with the selective concentrations of respective extracts. As shown in Figure 6, treatment of insulin‐resistant HepG2 cells with onion extracts decreased the expression levels of PTP1B, which was in accordance with their glucose uptake potential.

## 4 DISCUSSION

Onions are among the most highly consumed vegetables worldwide in which flavonoids and the phenolic acids are the predominating ones. It was reported that polyphenolic compounds, including flavonoids, are safe and nontoxic antioxidants. High dietary intake of natural phenolics has a strong correlation with longer life expectancy; reduced risk of developing some chronic disease, cancer, diabetes, and obesity; improved endothelial function; and blood pressure maintenance as suggested by various studies (Halliwell, 2007; Hodgson & Croft, 2006; Yan & Asmah, 2010).

In this study, we compared the 70% ethanol and water extracts of red and yellow onion peel cultivars for the TFC and TPC and found out that the level of TFC and TPC was higher in yellow onion cultivars than red onion in both 70% ethanol and water extracts. The data comply with the result suggested by the Cheng et al. (2013) and are contrary to the study undertaken by Albishi, John, Al‐Khalifa, and Shahidi (2013), Prakash, Singh, and Upadhyay (2007), and Shon, Choi, Kahng, Nam, and Sung (2004) on the content of TFC and TPC among cultivars. These authors used 50% methanol; a mixture of methanol, acetone, and water (7:7:6 v/v/v); and ethyl acetate for the extraction. However, we used 70% ethanol and water for extraction. Ethanol was suggested as the best extraction solvent for almond hull extraction (Pinelo, Rubilar, Sineiro, & Nunez, 2004). A comparative study by Jung, Seog, Choi, Park, and Cho (2006) on TPC and TFC on different solvent extracts of wild ginseng leaves showed higher content in ethanol extract than methanol and water. Further, Patil, Pike, and Hamilton (1995) showed that the onion contents differ greatly with location, growing stage, and soil type among 75 cultivars grown in Texas.

Antioxidant assay methods are based on different mechanisms, so single method to evaluate the antioxidant activity is not appropriate (Prior, Wu, & Schaich, 2005). In our study, we showed the antioxidant activity of onion in red and yellow onion cultivars in 70% ethanol and water extracts via DPPH radical and ABTS radical scavenging activity. These all extracts showed good activity against them. However, the YE extract showed relatively higher activity among others in both DPPH radical scavenging activity and ABTS radical scavenging activity with the IC_50_ value of 4.50 ± 0.06 and 6.64 ± 0.03 μg/mL, respectively. This may be due to the high level of TPC and TFC as suggested by Abdennacer, Karim, Nesrine, Mouna, and Mohamed (2015) and Pietta, Simonetti, and Mauri (1998). Yellow onion water extract had also shown greater antioxidant activity via DPPH radical scavenging activity and ABTS radical scavenging activity in accordance with the positive control. These data suggest that the peel can be used as natural antioxidants similar to the tea as suggested by Suzuki, Pervin, Goto, Isemura, and Nakamura (2016). The onion extracts had phenolic hydroxyl groups in the structure, and they have been recognized to function as electron or hydrogen donors (Shahidi, Janitha, & Wanasundara, 1992). According to Santas, Carbo, Gordon, and Almajano (2008) and Nuutila, Puupponen‐Pimiä, Aarni, and Oksman‐Caldentey (2003), flavonoids are the main compounds responsible for the antioxidant activity of onion peel.

Protein tyrosine phosphatase 1B (PTP1B) is an intracellular PTP localized on endoplasmic reticulum with its phosphatase domain oriented toward the cytoplasm and is expressed ubiquitously including in the classical insulin target tissues such as liver, muscle, and fat. Overexpression of PTP1B has been shown to inhibit signaling events both proximal and distal to the insulin receptor. Binding of insulin to insulin receptor leads to autophosphorylation of protein kinase. However, PTP1B interacts and removes tyrosine phosphates from insulin receptor. Further, PTP1B has the ability to dephosphorylate insulin receptor substrate proteins, thus attenuating and potentially terminating insulin signaling transduction (Zhang & Lee, 2003). It is reported that PTP1B‐knockout mice exhibit high level of insulin sensitivity and are also resistant to high‐fat diet‐induced obesity. These specific results suggest that PTP1B plays a decisive role in the T2DM. Therefore, particular PTP1B inhibitors may have therapeutic benefits to T2DM (Fukuda et al., 2010). Over activity of PTP1B leads to the pathogenesis of insulin resistance in the obese patient. So the main strategy of T2DM is the inhibition of PTP1B (Johnson, Ermolieff, & Jirousek, 2002; Klaman et al., 2000; Koren & Fantus, 2007). In this study, we compared the PTP1B inhibitory activity of RE, RW, YE, and YW extracts. All the extracts showed high inhibitory activity, YW being the best. YW extract showed the 10 times more potent activity than the positive control ursolic acid. Water extract had high activity than 70% ethanol extract against PTP1B. This recommends that water is the more appropriate solvent for extraction of compounds with PTP1B inhibitory activity from onion. Furthermore, in insulin‐resistant HepG2 cell model, these extracts decreased the expression level of PTP1B in a concentration‐dependent manner and enhanced the insulin‐stimulated glucose uptake. From this result, we can speculate that the onion extract contains insulin sensitizers, which could be the reported compounds like quercetin and its derivatives along with other components that are yet to be reported. Owing to higher toxicity of ethanol extracts of both onion cultivars compared to water extract in our MTT assay and higher activity of water extract, overall results suggest that the water extract of yellow and red onion peels can be used in the prevention and management of T2DM. In addition, less toxicity of water extract might be attributed to the presence of highly glycosylated compounds as glycosylation leads to high polarity and reduced toxicity. So it is worth to characterize the specific novel components in the water extract that solely enhanced the glucose uptake by increasing the sensitivity of HepG2 cells toward insulin in the present study.

α‐Glucosidase is secreted in the epithelium of small intestine, which acts as a key enzyme for carbohydrate digestion (converts disaccharides to monosaccharides). It has been recognized as a therapeutic target for the alleviation of postprandial hyperglycemia (Kim, Jeong, Wang, Lee, & Rhee, 2005). Thus, inhibitors can hinder the absorption of dietary carbohydrates followed by postprandial hyperglycemia suppression and could be powerful apparatus in easing hyperglycemia, and thus valuable for T2DM patients (Watanabe, Kawabata, Kurihara, & Niki, 1997). α‐Glucosidase inhibitors are commonly used as oral hypoglycemic agents. But long‐term use may cause flatulence, vomiting, and diarrhea (Hanefeld, 1998). There are numerous investigations regarding the discovery of α‐glucosidase inhibitors from natural products having a lower side effect (Fujita, Yamagami, & Ohshima, 2001; Gholamhoseinian & Fallah, 2009; Shim et al., 2003; Wang, Du, & Song, 2010). In Korea, guava leaf extract and Touchi‐extract have been approved as authorized health functional foods to improve postprandial hyperglycemia and in Japan, as Foods for Specified Health Use (FOSHU) (Kang et al., 2010).

In this comparative study, we evaluated the α‐glucosidase inhibitory activity of RE, RW, YE and YW. All extracts showed the higher inhibitory activity in comparison with positive control (acarbose). Among them, YE extract showed highest inhibitory activity against α‐glucosidase, which was 20 times better than the positive control. The second most active extract was YW. It was 12 times more potent than the positive control acarbose. The main target of diabetic therapy is to control fasting and postprandial hyperglycemia to maintain the blood glucose level. In comparison with fasting hyperglycemia, postprandial hyperglycemia is more strongly correlated with cardiovascular morbidity and mortality (Kang et al., 2010). Thus, YE and YW extracts may be beneficial in the management of diabetes.

Diabetes mellitus (DM) favors the production of AGEs. Formation and accumulation of AGEs in DM occur in rapid rate with other complications of cell injury with the increase in the production of reactive oxygen species that causes glucose auto‐oxidation and nonenzymatic protein glycosylation pathway reactions (Dzib‐Guerra et al., 2016). The important role played by AGEs in the pathogenesis of microvascular damage that includes nephropathy, retinopathy, neuropathy, and cataracts in DM is well recognized; similarly, the elevated levels of AGEs and their receptors have been detected in obese individuals (Merhi, Mcgee, & Buyuk, 2014). Thus, the compounds that have the ability of radical scavenging or metal chelation, also metabolites that can trap dicarbonyl species or break AGEs, are the appropriate candidate to limit or deduce the level of AGEs and related complications. In this study, 70% ethanol extract of both cultivars showed potent inhibitory activity on AGEs. YE and RE extracts have almost 5 times more strong activity than the positive control. Further, YW extract has double the inhibitory activity than the positive control (aminoguanidine). Harris et al. (2011) suggested the correlation between the total phenolic content and the anti‐AGEs activity. Our data comply with that, as YE and RE had the highest TPC, so as the anti‐AGEs activity.

To sum up, the comparative inhibitory activities of different extracts can be explained by their compositional differences. Phenolic and flavonoids were expected to be present in larger amount in the 70% ethanol extracts than the water extract, which is confirmed by our results. While comparing the antioxidant activity of onion peel between the different extracts, we found that the 70% ethanol extract had higher antioxidant activity than the water extracts. Additionally, Lee et al. (2014) reported the similar results while comparing the antioxidant activities of onion. Further, 70% ethanol extract showed better inhibitory activity on α‐glucosidase and AGEs than the water extract on both cultivars. This may be due to the presence of high levels of TPC and TFC like quercetin along with other bioactive compounds as suggested by Kim et al. (2011). Conversely, water extracts showed higher PTP1B inhibitory activity than the 70% ethanol extract. These data suggest that there are other more polar active compounds that are responsible for the specific inhibition of PTP1B activity. Due to the various side effects with the synthetic inhibitor, the current therapeutic effort has very limited success (Peyroux & Sternberg, 2006). Nutritional or herbal interventions based on the plant with high phenolic content represent a therapeutic approach with reduced risk of adverse effect as well as side effect and increase the patient compliance (Harris et al., 2011).

## 5 CONCLUSION

Overall, results indicate that the extract from peel of both red and yellow onions possesses a significant in vitro antioxidant and antidiabetic potential. The inhibitory activities of onion peel extract differed among red and yellow cultivars. At 2 μg/ml concentration, red onion peel extract displayed potent PTP1B inhibitory activity compared to yellow peel extract, while yellow onion had better α‐glucosidase inhibition. From this result, it may be concluded that red onion peel is superior in either quality or quantity of PTP1B inhibitors and/or insulin sensitizers to yellow onion peel. Similarly, among 70% ethanol and water as extracting solvents, 70% ethanol might be an appropriate for obtaining natural antioxidants. However, detail evaluation should be required to investigate the nature and amount of compounds in onion peel extract along with thorough in vivo studies.

## 6 LIMITATIONS

The present comparative study was conducted on extracts of only two onion cultivars (red and yellow) using two extracting solvents (70% ethanol and water). This is an in vitro study. Further in vivo study must be conducted to confirm our findings.

## CONFLICT OF INTEREST

The authors declare that there are no conflict of interests.

## ETHICAL STATEMENTS

This study does not involve any human or animal testing.
